# Childhood Obesity, Endothelial Cell Activation, and Critical Illness

**DOI:** 10.3389/fped.2020.00441

**Published:** 2020-08-05

**Authors:** Monique Radman, John McGuire, Jerry Zimmerman

**Affiliations:** Seattle Children's Hospital, Pediatric Critical Care, University of Washington, Seattle, WA, United States

**Keywords:** obesity, endothelial, activation, dysfunction, critical illness, inflammation, pediatric

## Abstract

Pediatric obesity is increasing in prevalence and is frequently an antecedent to adult obesity and adult obesity-associated morbidities such as atherosclerosis, type II diabetes, and chronic metabolic syndrome. Endothelial cell activation, one aspect of inflammation, is present in the early stages of atherosclerosis, often prior to the onset of symptoms. Endothelial activation is a pathological condition in which vasoconstricting, pro-thrombotic, and proliferative mediators predominate protective vasodilating, anti-thrombogenic, and anti-mitogenic mediators. Many studies report poor outcomes among obese children with systemic endothelial activation. Likewise, the link between childhood obesity and poor outcomes in critical illness is well-established. However, the link between obesity and severity of endothelial activation specifically in the setting of critical illness is largely unstudied. Although endothelial cell activation is believed to worsen disease in critically ill children, the nature and extent of this response is poorly understood due to the difficulty in measuring endothelial cell dysfunction and destruction. Based on the data available for the obese, asymptomatic population and the obese, critically ill population, the authors posit that obesity, and obesity-associated chronic inflammation, including oxidative stress and insulin resistance, may contribute to endothelial activation and associated worse outcomes among critically ill children. A research agenda to examine this hypothesis is suggested.

## Introduction

Childhood obesity is a worldwide epidemic, resulting in a significant predisposition to adult obesity and increased cardiovascular morbidity and mortality (1–3). Endothelial activation (EA) consists of endothelial cell (EC) dysfunction, destruction and impaired repair and represents, in part, a pathological imbalance between endothelium-derived contracting and relaxing factors (4, 5). More importantly, it is an early and often asymptomatic sign of atherosclerotic disease in obese patients (6). Early EA detection may identify children at risk of developing cardiovascular disease so that interventions can be implemented to prevent and/or reduce disease progression and to mitigate exaggerated acute EA in acute or critical illness.

## Normal Endothelial Function

Healthy endothelium responds to mechanical, neurogenic, and chemical signals with factors that regulate vascular tone, platelet aggregation, leukocyte adhesion and migration, mediator production, and smooth muscle cell proliferation (7). Endothelial vasoactive molecules dilate or constrict the microvasculature to balance tissue oxygen supply and metabolic demand, particularly in times of illness or injury.

Nitric oxide (NO), produced from the amino acid L-arginine through the action of constitutive endothelial NO synthase (eNOS) and cofactors such as tetrahydrobiopterin, is of central importance in all vascular beds (8). NO diffuses across the EC membrane to activate vessel wall smooth muscle cell guanylate cyclase to increase cyclic guanosine monophosphate concentrations with resultant smooth muscle relaxation. In healthy endothelium, eNOS is activated by adenosine, bradykinin, serotonin (produced during platelet aggregation), and vascular endothelial growth factor (VEGF) (stimulated by hypoxia) (9). Endothelium produced prostacyclin also mediates vasodilation (10).

Normal endothelium also generates vasoconstrictors including endothelin-1 (ET-1) and the conversion of EC-secreted angiotensin I to angiotensin II (ATII) by angiotensin-converting enzyme (ACE).

NO maintains endothelial quiescence by inhibiting inflammation, cellular proliferation, and thrombosis (11). Mechanically, laminar blood flow appears to enhance EC survival by suppressing apoptosis. Conversely, turbulent flow can trigger EC division (12–14). The endothelial glycocalyx is a network of membrane-bound proteoglycans and glycoproteins covering the endothelium lumen that integrates endothelium- and plasma-derived soluble molecules. Over the past decade, the role of the glycocalyx in vascular mechanotransduction, hemostasis, signaling, and blood cell–vessel wall interactions has been elucidated (15). Neurogenically, endothelial function is modulated by the “gateway reflex,” or neural circuits regulating entry of immune cells to the CNS by modulating the vascular EC barrier. The gateway reflex phenomenon also contributes to recruitment of immune cells to other tissues (16).

## Endothelial Activation

While EA is often a normal immune response to insult or injury, it may become pathologic. This switch is characterized by three major components: EC dysfunction, destruction, and impaired repair. Based on the data summarized below from studies in obese, asymptomatic and obese, critically ill populations, it is plausible that obesity and obesity-associated chronic inflammation, oxidative stress, and insulin resistance (IR) may intensify EA and, in turn, contribute to worse outcomes among obese, critically ill children (17). Extreme nutrient excess may cause obese adipocyte cell death resulting in cytokine and fatty acid release. These may be sensed by inflammatory kinases, or Toll-like receptors (18). Kinases downstream of these receptors [c-jun N-terminal kinase (JNK), protein kinase R (PKR)] can, in turn, become activated and inhibit insulin signaling via phosphorylation of insulin receptor substrate 1 (IRS-1), thereby blocking insulin action and further inhibiting energy metabolism (19–21).

Insulin modulates vascular tone by regulating expression of eNOS gene in ECs, mediated by the activation of phosphatidylinositol-3 kinase (PI-3K). Activation of protein kinases such as PKR, as in IR, may inhibit PI-3K activity and eNOS expression (22). Superimposition of critical illness such as sepsis, acute respiratory distress syndrome (ARDS), multiple-organ dysfunction syndrome (MODS), trauma, and cardiopulmonary bypass (CPB) for cardiac surgery may compound the effects of obesity by further overwhelming these pathways (17).

### Endothelial Cell Dysfunction

EC dysfunction constitutes a shift to a predominant generation of vasoconstrictors, including ET-1 and ATII and enhanced reduction-oxygenation (redox) signaling, resulting in reactive oxygen species (ROS), or free radicals that cause oxidative stress (5, 18). Superoxide anion (${\text{O}}_{2}^{-}$) is generated and converted to hydrogen peroxide (H_2_O_2_) by superoxide dismutase (23, 24). H_2_O_2_ stimulates gene transcription and protease activation (25). Normally a key enzyme in NO-associated processes, eNOS may generate ROS like H_2_O_2_ and ${\text{O}}_{2}^{-}$ in the absence of L-arginine and tetrahydrobiopterin, respectively. This shift is referred to as “eNOS uncoupling,” a hallmark of pathologic EA (8). Obese patients live in a persistent state of relatively high ROS production and, consequently, a perpetual state of EA (26). In critical illness, decreased substrate delivery, and hypoxemia interrupt oxidative phosphorylation and more ROS may be produced, leading to further EA and inflammation (27–30).

### Endothelial Cell Destruction

In the setting of cardiovascular risk factors, EC dysfunction may progress to destruction and loss of vessel endothelial integrity. For example, after ischemia/reperfusion, ECs swell and detach from the basement membrane (31). These cells suffer oxidative stress, leukocytes adhere/transmigrate, and vascular permeability increases (32–34). In animal models, ischemia/reperfusion leads to shedding of glycosaminoglycan chains and reduced glycocalyx thickness (35).

EC apoptosis results in circulation of both whole ECs and EC microparticles in the periphery as well as the coronary arteries. They are indicative of atherosclerosis or other inflammation-associated endothelial damage and have been independently quantified in both obese and critically ill individuals (36–40). The nature and extent of EC damage in obese, critically ill patients needs focused study.

### Impaired Endothelial Cell Repair

Endothelial repair in response to injury or inflammation is crucial to vascular health (41). The key player in endothelial repair is the circulating endothelial progenitor cell (EPC). After release from the bone marrow, EPCs can differentiate into mature ECs. Paradoxically, EPCs are released from the bone marrow in response to NO. Among patients with impaired NO production (e.g., obesity), EA is further exacerbated (42). Interventions aimed at ameliorating cardiovascular risk factors such as exercise and statins increase EPC release and differentiation (39, 43, 44). Obesity interferes with both EPC mobilization and EPC function, and the number of risk factors for coronary artery disease is inversely related to the number and migratory activity of EPCs (39). Circulating EPCs, in an inflammatory milieu, can also differentiate into myeloid cells, such as macrophages, a key component of EA and atherosclerosis (45). It follows that a combination of obesity (chronic nutrient excess, metabolic pathway overload, and inflammation) and critical illness (acute inflammation) may exaggerate macrophage differentiation and pathologic EA.

### Measuring Endothelial Function

The most commonly used non-invasive research method for assessing endothelial function is brachial artery diameter measurement using ultrasound before and after several minutes of blood flow occlusion. This change in arterial diameter is referred to as flow-mediated vasodilation (FMD), and the increase in blood flow as “reactive hyperemia.” When performed properly, this method correlates strongly with coronary artery endothelial function (46, 47).

Direct products of EA can be measured but currently have limited clinical application due to lack of specificity, assay availability, and performance variability. These include measures of NO bioavailability, adhesion molecules, inflammatory cytokines, mediators of thrombosis, and markers of endothelial damage and repair (EPCs). Despite the lack of mechanistic specificity, these serum markers remain the most frequently used research measures of endothelial function in inflammatory states including critical illness and obesity (48–50).

## Childhood Obesity and Inflammation

Childhood obesity is particularly problematic as it independently contributes to adult morbidity (2, 51–53). Unlike obese adults, EA in obese children often silently affects the microcirculation rather than manifesting in the macrocirculation. Obese children rarely have atherosclerotic lesions and many have not yet developed type II diabetes or hypertension (46). Additionally, puberty-related pro-oxidative and pro-inflammatory changes and relative IR may impact the natural history of EA in obese children (54–56).

Obesity results in “metaflammation,” a chronic, low-grade inflammatory response to excess energy substrate by metabolic cells including adipocytes, hepatocytes, myocytes, pancreatic islets of Langerhans, and astrocytes, and neurons (57). Immune cells activated by metabolic cell inflammatory signaling exacerbate tissue inflammation.

## Obesity-Associated Endothelial Activation

EA is an early and often asymptomatic sign of atherosclerotic disease in obese children and adults (46). Many of the pro-inflammatory and pro-atherogenic markers associated with vascular disease in adults have also been demonstrated in obese, otherwise healthy children.

### Obese, Asymptomatic Children

Although atherosclerotic lesions are rare in children, obesity has a major impact on the development of EA and atherosclerosis. Schlager et al. measured microvascular function in obese, normotensive children with FMD. Compared to non-obese controls, obese children had higher peak perfusion during hyperemia and longer recovery time, indicative of impaired endothelium-dependent microcirculation vasodilation following ischemia and can be interpreted as an early sign of EA in obese children (58). In contrast, a previous study performed in lean, overweight and obese, hypertensive adolescents found no significant differences in endothelium-dependent microvascular reactivity among the three groups, suggesting that a blood pressure rise likely precedes endothelium-dependent microvascular function deterioration in juvenile essential hypertension pathogenesis (59). Thus, while obesity is strongly associated with hypertension, it is unclear whether EA precedes or is a consequence of other processes.

Chronic, low-grade inflammation, and resultant oxidative stress trigger early vascular damage in obese children. Associating markers of inflammation and oxidative stress with functional assessments of the vasculature provides an opportunity to understand mechanisms that may be targeted to mitigate cardiovascular disease. Studies have shown an inverse correlation between FMD and inflammatory markers [C-reactive protein (CRP), Interleukin (IL)-6, and Intercellular adhesion molecule (ICAM)-1 levels] in obese, otherwise healthy children (60–62).

### Critical Illness

**Figure 1:**
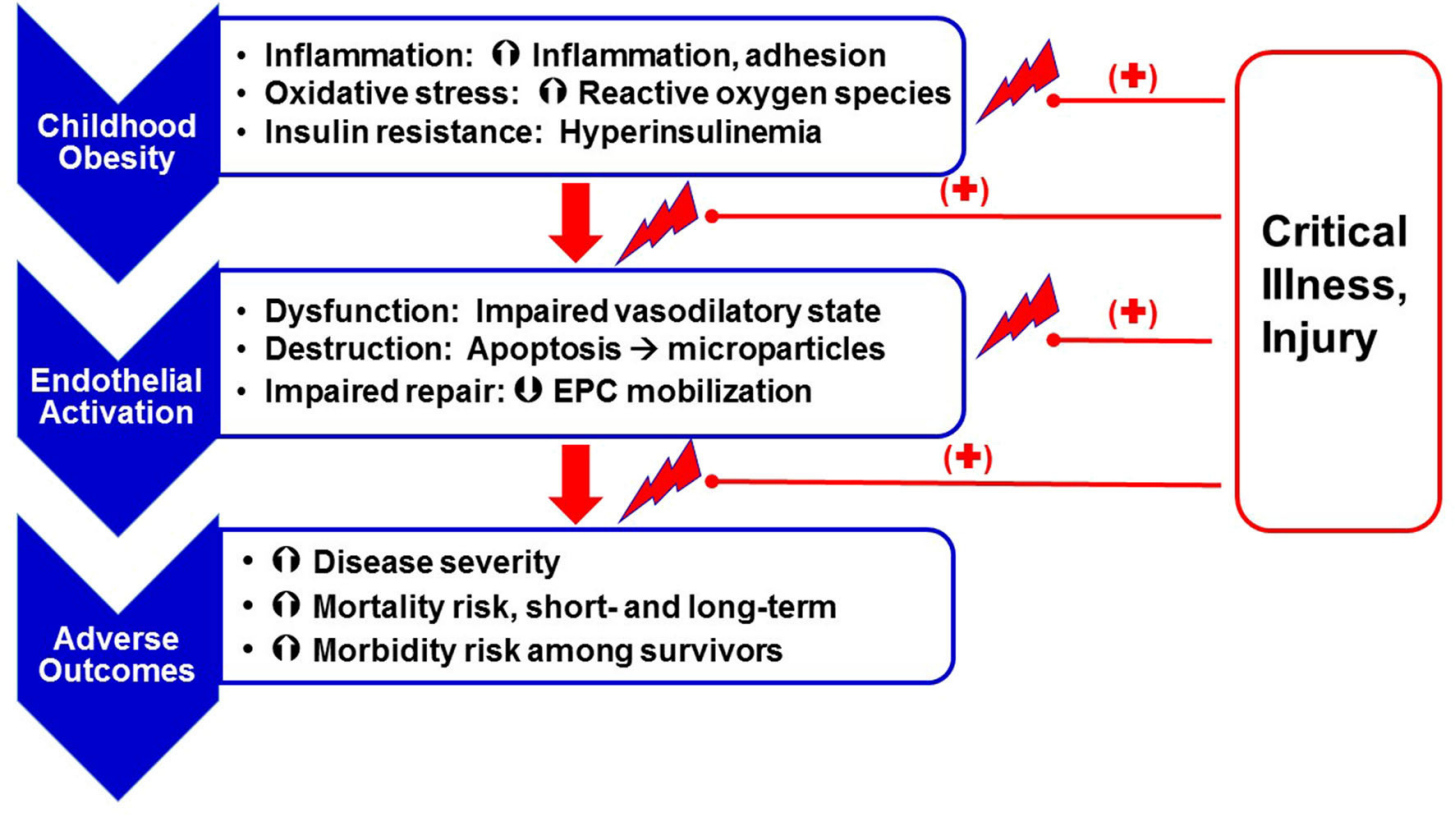
Obesity as a risk factor for chronic endothelial (microvascular) stress and adverse outcomes following critical illness. Obesity, secondary to excess nutrient intake, leads to a state of chronic inflammation [increased expression of pro-inflammatory mediators like Interleukin (IL)-6 and IL-8 and adhesion molecules like Intercellular adhesion molecule (ICAM)-1, Vascular cell adhesion molecule (VCAM) and E-selectin, enhanced oxidative stress (superoxide anion and hydrogen peroxide production), and insulin resistance]. These obesity-related effects lead to endothelial activation, including endothelial cell dysfunction (decreased production of vasodilatory mediators such as nitric oxide and prostaglandins E and I, and increased release of endothelin-1), cell destruction (resulting in release of endothelial microparticles), and impaired repair, in part, as a result of modulated endothelial progenitor cell mobilization. Obesity-related endothelial activation is associated with worse clinical outcomes. Superimposition of critical illness on a chronic state of endothelial activation further facilitates risk for adverse outcomes.

Several studies reported associations between poor outcomes and EA in critically ill children (63–67). Likewise, the link between childhood obesity and poor outcomes in critical illness is well-established (68–71). However, the link between obesity and EA severity specifically in the setting of critical illness is largely unstudied. Although EA may worsen disease in critically ill children, the nature and extent of this response is poorly understood due to the difficulty in measuring EC dysfunction and destruction. Based on the data available in the obese, asymptomatic population and the obese, critically ill population, it follows that worse EA-related outcomes in the PICU may, at least in part, be associated with obesity and associated chronic inflammation, oxidative stress and IR (Figure 1).

ECs act as targets and amplifiers of the cytokine signaling that characterizes critical illness including sepsis, ARDS, MODS, and CPB. The etiology of progression to MODS in the ICU is often unknown. However, the presence of ECs within every organ system suggests a pathophysiologic role (72). Adhesion molecules such as E-selectin are significantly higher in septic children with >3 failed organ systems compared to those with ≤ 3 failed organ systems, suggesting a role in EA (73). Pro-inflammatory markers, specifically IL-1, IL-6, and tumor necrosis factor, activate ECs to produce and express adhesion molecules (ICAM-1, E-selectin) to promote leukocyte motility and adhesion in the systemic inflammatory response (17). Derangements in endothelial and inflammatory biomarkers (higher VEGF, thrombomodulin, CRP, IL-6, IL-8) were shown in children upon presentation to the ICU with extrapulmonary sepsis with and without ARDS (74). Lastly, obesity-related EA among critically ill children with malignancies is largely unstudied. When not acutely ill, children with malignancies demonstrate worse FMD compared to controls. Lower FMD scores correlated with serum biomarkers of EA and increased waist circumference, pointing to adiposity as a potential exacerbating factor in critical illness (75).

### Congenital Heart Disease and Cardiopulmonary Bypass

The impact of obesity on endothelial function and long-term cardiovascular health may be particularly important to children with preexisting acquired or congenital heart disease (CHD). Systemic and pulmonary vascular EA are well-documented in CHD and contribute to a lifetime of increased risk of mortality and morbidity as poor exercise capacity, ventricular dysfunction, and development of thromboembolic disease (76–78). A recent study estimated an overweight/obesity prevalence of 30% among single ventricular patients 5 years after undergoing a Fontan (79). Importantly, increased adiposity is independently associated with worse endothelial function and worse functional outcomes after the Fontan operation (80, 81). Understanding the relationship between vascular function and functional outcomes and the specific role of obesity is an important next step.

Low cardiac output syndrome (LCOS) following CPB in repair of CHD is characterized by a transient decrease in systemic perfusion secondary to myocardial dysfunction and is a manifestation of EA. LCOS contributes to postoperative morbidity including prolonged mechanical ventilation and hospitalization, increased risk of infection, and long-term adverse neurologic sequelae (82). Factors involved in LCOS include activation of inflammatory and complement cascades, altered blood flow, and ischemia-reperfusion injury (83).

CPB increases vasoconstrictors that contribute to vascular reactivity and decreased microcirculatory flow and result in myocardial injury. Additionally, CPB-induced EA shifts the anticoagulant phenotype to procoagulant. EC surface tissue factor expression increases, leading to deposition of fibrin in the microcirculation. Procoagulant properties are further propagated by the simultaneous downregulation of thrombomodulin (84). Lastly, CPB-associated EA stimulates surface protein expression that facilitates leukocyte-EC interactions. P-selectin release facilitates the strong bond between neutrophil surface integrins and adhesion molecules on the EA surface. This cascade precedes neutrophil infiltration into the perivascular tissue and, ultimately, the production and release of ROS and proteases that mediate cellular and end-organ damage (85).

## Therapies

Determining nutritional goals for obese, critically ill children remains challenging, as commonly used equations to estimate caloric needs frequently under- or overestimate energy requirements (86). Overfeeding consequences include prolonged mechanical ventilation and hospitalization and hyperglycemia (87). The American Society for Parenteral and Enteral Nutrition recommends direct measurement of resting energy requirements utilizing indirect calorimetry. However, patients must be at “steady state” (no ongoing titration of oxygen and/or inotropes) to achieve accurate measurements, making this recommendation difficult to implement (88). Treatment options specifically for the obese child with presumed chronic inflammation and EA are outlined below. These treatments have the potential to reverse chronic EA and/or dampen acute EA in the setting of critical illness.

### Exercise

Physical activity is promising as a therapeutic tool in obese children. Multiple studies have shown an improvement in FMD accompanied by a decrease in BMI with exercise interventions (89–91). Moreover, FMD may improve even in the absence of fat loss or decrease in BMI (92). These findings are likely explained by increased NO bioavailability due to exercise-induced shear stress (93). Importantly, exercise interventions that improved FMD without weight loss failed to decrease markers of inflammation and/or oxidative stress (94), underscoring the central role of fat mass in the inflammatory cascade. Watts et al. highlighted the importance of long-term, uninterrupted exercise in obese children by demonstrating a reversal of exercise-associated improvement in FMD after just 6 weeks of inactivity (95). In summary, an exercise program, even without dietary modifications, can improve baseline endothelial function, and may decrease vulnerability in the event of critical illness among overweight/obese children (89).

### Statins

Two large, adult trials reported improvement in endothelial function with the use of cholesterol-lowering therapy (96, 97). HMG-CoA reductase inhibitors (statins) exhibit antioxidant, anti-inflammatory, and NO restorative properties (98), and beneficial effect on endothelial function has been shown in a broad range of patients (99–101). Statin therapy is now a first-line pharmacologic intervention for children with severe dyslipidemias failing treatment with diet and exercise alone (102). Statins also improve endothelial function in hypertension and hypercholesterolemia and may be useful alone or in combination with other agents.

### L-Citrulline

L-citrulline is a naturally occurring amino acid and first intermediate in the urea cycle. Once produced, citrulline is transported from the mitochondria to the cytoplasm and converted to arginine, the precursor for NO. In multiple observational and clinical studies, plasma levels of citrulline, and arginine drop precipitously in CPB and do not recover for up to 48 h. In a recent study, CPB significantly decreased several urea cycle intermediates and NO metabolites after repair of unrestrictive ventricular septal defect and atrioventricular septal defect (103). In a phase IIb trial, patients receiving intravenous L-citrulline showed reduced duration of mechanical ventilation, inotropic needs, and ICU stay (104). Further study of associations between L-citrulline administration and endothelial function is needed in this population.

### Preservation of The Glycocalyx

The endothelial glycocalyx is responsible for maintaining homeostasis of intravascular flow and dynamics. It is affected in inflammation and hyperglycemia and has a central role in capillary leak syndrome, or “endothelial failure syndrome,” particularly in sepsis (105). Sulodexide, a mix of glycosaminoglycan precursors, inhibits matrix metalloproteinases, and IL-6 while stimulating lipoprotein lipase activity and modulating the coagulation-fibrinolysis balance. In a recent animal sepsis study, sulodexide accelerated glycocalyx regeneration with decreased vascular permeability, and improved survival (106). Additionally, maintaining adequate levels of plasma proteins, particularly albumin, may promote glycocalyx repair (107, 108). Lastly, evidence suggests that resuscitation fluid volume and composition may impact glycocalyx stability and the extent of end-organ injury (109, 110). Further studies are needed to determine which therapies have direct effects on glycocalyx integrity and its relationship to ICU outcomes.

### Other Treatments That May Regulate Vascular Function

Endothelin receptor antagonists (ERAs) have a high potential in the treatment of hypertension and renal diseases such as diabetic nephropathy by blunting endothelium-dependent vasoconstriction (111). The evidence for the potential benefits of ERAs is limited in children, and more data is needed.

Phosphodiesterase inhibitors (PDEIs) have promise as a treatment in specific pediatric populations. A recent phase III clinical trial demonstrated improved exercise performance after treatment with a PDEI among children who had undergone a Fontan procedure (112). Further investigation is needed to determine the effects of chronic treatment.

Other novel therapies with limited, but promising data that are outside of the scope of this review include metabolic therapies such as thiamine, vitamin C, tight glycemic control with insulin, lipid/triglyceride modulation, and periodic whole body acceleration.

## Conclusions

The endothelium is crucial in maintaining vascular homeostasis in health and allostasis in critical illness. Under stress, ECs become activated, initiating inflammation, followed by new cell surface protein gene expression. While this response represents an attempt to neutralize infection and injury, it can be pathologic. Patients with baseline chronic inflammation, such as obese children, may be at risk for exaggerated EA and associated end-organ injury, morbidity, and mortality. However, this paradigm has not been adequately investigated among obese, critically ill children, and represents a current gap in pediatric research.

The relationship between obesity, EA, and outcomes of critically ill children is ripe with research potential. Further understanding of the relationships between weight loss and inflammation may inform specific diet and lifestyle modifications to prevent and/or reverse obesity-related EA. While EA is associated with higher illness severity and adverse outcomes, the severity and mechanisms involved are difficult to assess directly in critically ill children. Microcirculation markers such as capillary refill time frequently lack correlation with macrocirculation measures such as blood pressure or peripheral pulses (113). Additionally, studies on biomarkers of EA such as proteins or damaged ECs or EC particles continue to be an area of much needed future study.

## Author Contributions

MR, JM, and JZ contributed to the focus and design of the review. MR wrote the first draft of the manuscript. All authors contributed to manuscript revisions, read, and approved the submitted version.

## Conflict of Interest

The authors declare that the research was conducted in the absence of any commercial or financial relationships that could be construed as a potential conflict of interest.
